# Association of lncRNA H19 polymorphisms with cancer susceptibility: An updated meta-analysis based on 53 studies

**DOI:** 10.3389/fgene.2022.1051766

**Published:** 2022-12-14

**Authors:** Yingying Yuan, Yachun Wang, Xiaodong Niu, Yungang Han, Wenbo Li, Meijin Cheng, Zheng Li, Jiao Tan, Yue Zhao, Wei Wang

**Affiliations:** Department of Clinical Laboratory, Henan Province Chest Hospital, Zhengzhou University, Zhengzhou, China

**Keywords:** lncRNA, H19, cancer susceptibility, polymorphism, meta-analysis

## Abstract

The association between polymorphisms in lncRNA H19 and cancer susceptibility remains to be inconsistent. This study aimed to provide a more precise estimation of the relationship between lncRNA H19 polymorphisms and the risk of cancer based on all available published studies. 53 studies encompassing 32,376 cases and 43,659 controls were included in our meta-analysis by searching the Pubmed, Embase, Web of Science, WanFang, and China National Knowledge Infrastructure databases. Pooled ORs and their 95% CIs were used to estimate the strength between the SNPs in H19 (rs217727, rs2839698, rs2107425, rs3024270, rs2735971, rs3741216, and rs3741219) and cancer susceptibility. The results showed that H19 rs2839698 polymorphism was associated with increased cancer risk in all participants under three genetic models. However, no significant association was identified between the other six SNPs as well as an overall cancer risk. Stratification by ethnicity showed that rs2839698 mutation indicated to be an important hazardous factor for the Asian population. While rs2107425 mutation had a protective effect on the Caucasian population. Stratification by cancer type identified that rs217727 mutation was linked to increased susceptibility to oral squamous cell carcinoma, lung cancer, and hepatocellular carcinoma; whereas rs2839698 mutation was associated with an elevated risk of hematological tumor and digestive system tumor (*p* < 0.05). Besides, the rs2735971 mutation was connected with the digestive system tumor. In summary, the rs217727, rs2839698, rs2107425 and rs2735971 polymorphisms in H19 have associations with cancer susceptibility.

## 1 Introduction

Cancer is a serious disease affecting human health, which ranks as the leading cause of mortality worldwide (Dessale et al., 2022). It was estimated that there were about 19.3 million new cases and 10 million cancer-related deaths in 2020 (Sung et al., 2021), which is an important barrier to increasing life expectancy. There are many causes of the tumor, such as individual hereditary, chronic inflammation, unhealthy lifestyles, environmental pollution, and so on (Bade and Dela Cruz, 2020; Dugué et al., 2022). Among them, the role of genetic inheritance was widely accepted (Adolf et al., 2022). Numerous genome-wide association studies (GWAS), as well as case-control studies, have been performed in the last few decades to detect the association of thousands of genes and their mutation with cancer risk and prognosis (Lin et al., 2021; Yang et al., 2021). The long non-coding RNAs (lncRNAs) gene has attracted extensive attention for its critical role in carcinogenesis.

lncRNA, larger than 200 nts, is one of the most important members of the non-coding RNA family, which regulates gene expression through various mechanisms, such as inducing chromosome refactoring, synthesizing endogenous small interfering RNA, and changing protein localization (Bridges et al., 2021). Although only more than 5,000 lncRNAs have been identified, they play important roles in cell regulation at the transcriptional and post-transcriptional levels and are involved in multiple biological processes, including cell cycle, proliferation, and apoptosis (Xia et al., 2022).

LncRNA H19, located on chromosome 11p15.5, belongs to the human imprinted gene and contains five exons as well as four introns (Chen and Sima, 2019). It has been shown to be over-expressed in multiple cancers, such as lung cancer (Zhao et al., 2021), osteosarcoma (Zhao and Ma, 2018), prostate cancer (Singh et al., 2021), breast cancer (Chen et al., 2022), and so on. Given the oncogenic roles of H19 and the function of gene variation in regulating tumor growth and prognosis, increasing studies have been performed to explore the possible role of H19 SNPs in cancer susceptibility (Qin et al., 2019; Cao et al., 2020). For instance, Wu et al. conducted a study and found that H19 polymorphism rs2839698 might influence the susceptibility to hepatocellular carcinoma (HCC) (Wu et al., 2019). However, another study conducted by Li et al. showed that H19 polymorphism rs2839698 was not associated with neuroblastoma susceptibility (Li et al., 2019). In that case, though considerable studies have been performed, consistent results have not been obtained due to inadequate statistical power, ethnic differences, different genotyping techniques, and other reasons. In addition, twenty-four new relevant studies have emerged in the past 2 years. Therefore, this updated systematic review and meta-analysis of all eligible studies aimed to explore a more precise understanding of the association between lncRNA H19 SNPs and cancer risk.

## 2 Materials and methods

### 2.1 Search strategy

We searched the Pubmed, Embase, Web of Science, WanFang, and China National Knowledge Infrastructure (CNKI) databases up to May 2022 for relevant articles using the following terms: “lncRNA H19 OR long non-coding RNA H19 OR H19”, “cancer OR carcinoma OR tumor OR malignancy”, and “SNP OR polymorphism OR variant OR mutation”. Besides, we retrieved articles by reading the abstracts, and full articles published in English or Chinese without language restrictions. The references for each retrieved article were also investigated carefully for getting related studies.

### 2.2 Selection criteria

The studies included in our meta-analysis should meet the following criteria: 1) to evaluate the association between the H19 polymorphism and cancer risk; 2) case-control study or cohort study; and 3) genotype frequencies of case and control groups could be obtained to calculate the odds ratio (OR) and 95% confidence interval (CI). We excluded reviews, letters, meta-analyses, conference papers, and other studies that were short of sufficient genotyping data.

### 2.3 Data extraction

The information collection was conducted by two investigators independently, and a final agreement was reached by discussing with the third person when there were divergences. The following information was collected from both cases and controls groups: the first author’s name, publication year, country, ethnicity, cancer type, source of the control group, genotyping method, and evidence of Hardy-Weinberg equilibrium (HWE) in the control group.

### 2.4 Statistical analysis

HWE was evaluated based on the χ2 test in control subjects. Pooled ORs and their 95%CIs were used to estimate the strength between the seven SNPs in H19 (rs217727, rs2839698, rs2107425, rs3024270, rs3741216, rs3741219, and rs2735971) and cancer susceptibility in the following genetic models: allele model, recessive model, dominant model, homozygous model, and heterozygous model. Subgroup analysis was calculated concerning ethnicity, cancer types, sources of control, HWE status, and genotyping methods. Cochran’s Q test and I^2^ statistic were used to calculate heterogeneity (Tsironikos et al., 2022). When no heterogeneity exists (the I^2^ value <50% or *p* > 0.05), the fixed-effect model was performed. Otherwise, a random-effect model was applied. Furthermore, sensitivity analyses were performed to examine the stability of the results. Egger’s test and Begg’s funnel plots were used to assess the publication bias (Zhang et al., 2022). *p* > 0.05 and symmetrical funnel plots indicate no bias exits.

## 3 Results

### 3.1 Characteristics of included studies

**FIGURE 1:**
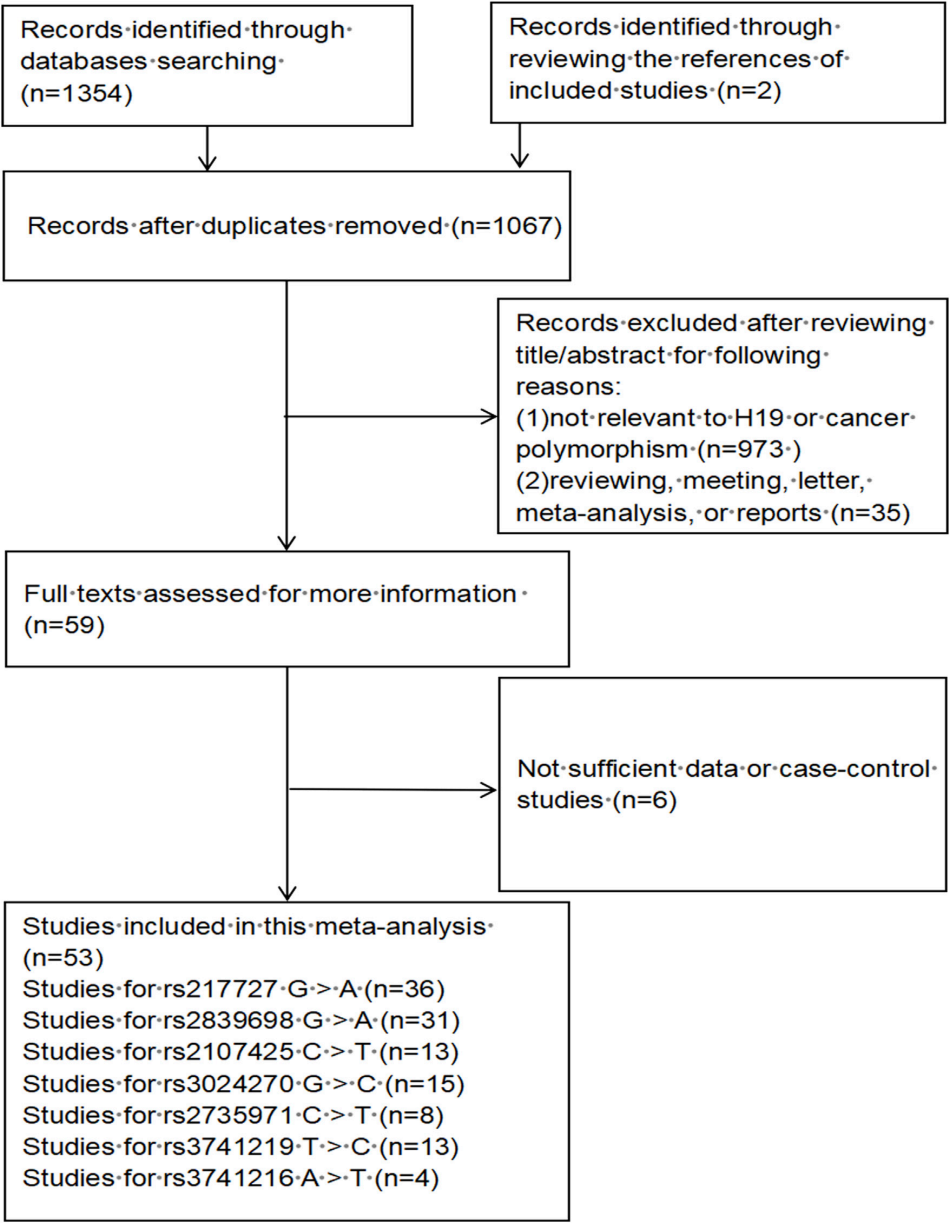
The flow diagram of this meta-analysis.

A total of 1,067 publications were preliminarily identified based on our search strategy using different search term combinations. All the abstracts and articles were scrutinized following the selection process (Figure 1). In the end, 53 studies encompassing 32,376 cases and 43,659 controls were incorporated in our meta-analysis, of which 24 studies were updated recently. The characteristics of the 53 studies are shown in Supplementary Table S1. Among the 53 studies, 45 studies were conducted in Asian populations, 6 studies in Caucasian populations, and 2 studies in African populations; 26 studies using the TaqMan genotyping method, 13 studies using PCR-RFLP, 6 studies using MassARRAY, others using KASP, Illumina and so on; the control group of 34 studies were population-based (PB), 19 studies were hospital-based (HB).

### 3.2 Meta-analysis results of rs2839698 polymorphism

**FIGURE 2:**
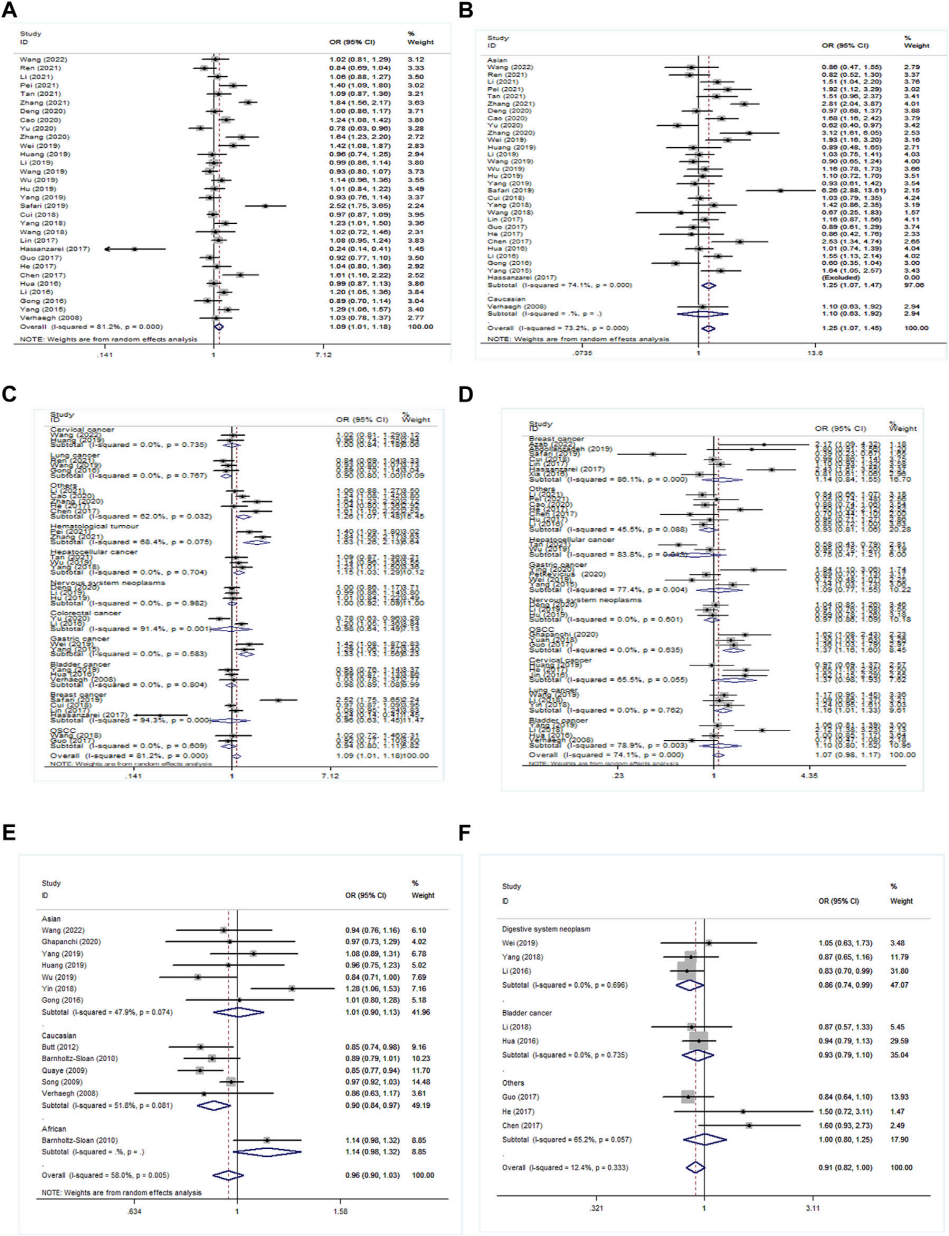
Forest plot for the association between H19 polymorphisms and cancer susceptibility. **(A)** Forest plot for rs2839698 polymorphism in the overall population, A vs G; **(B)** in ethnicity subgroup, AA vs GG; **(C)** and in cancer type subgroup, A vs G; **(D)** for rs217727 polymorphism in cancer type subgroup, AA + AG vs GG; **(E)** for rs2107425 polymorphism in ethnicity subgroup, T vs C; **(F)** for rs2735971 polymorphism in cancer type subgroup, TC vs CC.

Thirty-one studies investigated the association of rs2839698 polymorphism with cancer susceptibility. A hazardous effect on cancer development was observed in the H19 rs2839698 polymorphism under allele model (OR = 1.09, 95% CI = 1.01–1.18, *p* = 0.034); dominant model (OR = 1.11, 95% CI = 1.01–1.21, *p* = 0.029); and homozygous model (OR = 1.25, 95% CI = 1.07–1.46, *p* = 0.004) in all participates (Supplementary Table S2; Figure 2A).

Stratification by ethnicity identified that H19 rs2839698 mutation significantly increased cancer risk in the Asian population under allele model (OR = 1.09, 95% CI = 1.01–1.19, *p* = 0.035), dominant model (OR = 1.12, 95% CI = 1.02–1.22, *p* = 0.020) and homozygous model (OR = 1.26, 95% CI = 1.07–1.47, *p* = 0.004) (Supplementary Table S2; Figure 2B). Stratification by cancer type identified that H19 rs2839698 mutation increased the risk of digestive system neoplasm under allele model (OR = 1.14, 95% CI = 1.01–1.30, *p* = 0.040); recessive model (OR = 1.31, 95% CI = 1.02–1.69, *p* = 0.03); and homozygous model (OR = 1.33, 95% CI = 1.03–1.74, *p* = 0.03). Disease-specific meta-analysis was performed on hepatocellular cancer (HCC), gastric cancer (GC), colorectal cancer (CRC), and other digestive system neoplasms. The analysis indicated that rs2839698 mutation increased the risk of HCC and GC rather than CRC (Supplementary Table S2; Figure 2C). Furthermore, the significant association between the rs2839698 polymorphism and hematological tumor was demonstrated in all genetic models (*p* < 0.05) (Supplementary Table S2; Figure 2C). In analyses according to the source of control and genotyping methods, significant associations were observed between rs2839698 G>A and cancer susceptibility in PB and TaqMan (Supplementary Table S2).

### 3.3 Meta-analysis results of rs217727 polymorphism

Thirty-six studies were selected to estimate the association of the rs217727 SNP with the risk of cancer. No significant association was indicated for rs217727 through the pooled risk estimation in all genetic models (Supplementary Table S3).

In the further subgroup analysis by cancer type, we identified that H19 rs217727 mutation increased oral squamous cell carcinoma (OSCC) risk under all models (allele model: OR = 1.31, 95% CI = 1.14–1.50, *p* < 0.001; recessive model: OR = 1.67, 95% CI = 1.04–2.67, *p* = 0.034; dominant model: OR = 1.37, 95% CI = 1.16–1.60, *p* < 0.001; homozygous model: OR = 1.89, 95% CI = 1.19–2.99, *p* = 0.007; heterozygote model: OR = 1.27, 95% CI = 1.07–1.51, *p* = 0.005). The same association with increased lung cancer (LC) risk was also observed in four models (allele model: OR = 1.16, 95% CI = 1.06–1.27, *p* = 0.002; recessive model: OR = 1.31, 95%CI = 1.03–1.66, *p* = 0.028; dominant model: OR = 1.16, 95% CI = 1.01–1.33, *p* = 0.031; homozygous model: OR = 1.38, 95%CI = 1.14–1.67, *p* = 0.001). While the variant A allele of the rs217727 polymorphism resulted in significantly decreased risk for HCC in recessive model (OR = 0.73, 95% CI = 0.54–1.00, *p* = 0.048) and homozygous model (OR = 0.68, 95% CI = 0.49–0.93, *p* = 0.017) (Supplementary Table S3; Figure 2D). In the subgroup of genotyping methods, rs217727 was linked with increased cancer risk through the PCR-RFLP method (*p* < 0.05, Supplementary Table S3). However, no significant results were detected in the subgroup analysis by ethnicity and source of control.

### 3.4 Meta-analysis results of rs2107425, rs2735971, rs3024270, rs3741219 and rs3741216 polymorphisms

The association between the H19 rs2107425, rs2735971, rs3024270, rs3741219, and rs3741216 polymorphisms and cancer risk were separately examined in 13 studies, 8 studies, 15 studies, 13 studies, and 4 studies. We did not find any significant association between these five SNPs and cancer risk in all participants. However, in subgroup analysis stratified by ethnicity, cancer type, genotyping methods, and source of control, these SNPs were related to cancer susceptibility except for rs3741216.

The analysis of rs2107425 indicated that a variant T allele decreased cancer risk in the Caucasian population under the allele model (OR = 0.90, 95% CI = 0.84–0.97, *p* = 0.006), dominant model (OR = 0.84, 95% CI = 0.75–0.94, *p* = 0.003) and heterozygote model (OR = 0.82, 95% CI = 0.72–0.94, *p* = 0.003) (Supplementary Table S4; Figure 2E).

For rs2735971, this locus was linked with reduced cancer risk to digestive system neoplasm under three genetic models (allele model: OR = 0.88; 95% CI = 0.78–0.98, *p* = 0.021; dominant model: OR = 0.85, 95% CI = 0.74–0.98, *p* = 0.024; and heterozygote model: OR = 0.86, 95% CI = 0.74–0.99, *p* = 0.034) (Supplementary Table S4; Figure 2F). Additionally, in subgroup analysis by genotyping method, feebly positive results were shown in the TaqMan group for the rs3024270 locus. While the subgroup analysis stratified by the source of the control group indicated that rs3741219 increased cancer susceptibility in the HB control population (Supplementary Table S5). No significant association existed in other subgroups.

### 3.5 Detection of heterogeneity, sensitivity analysis, and publication bias

**FIGURE 3:**
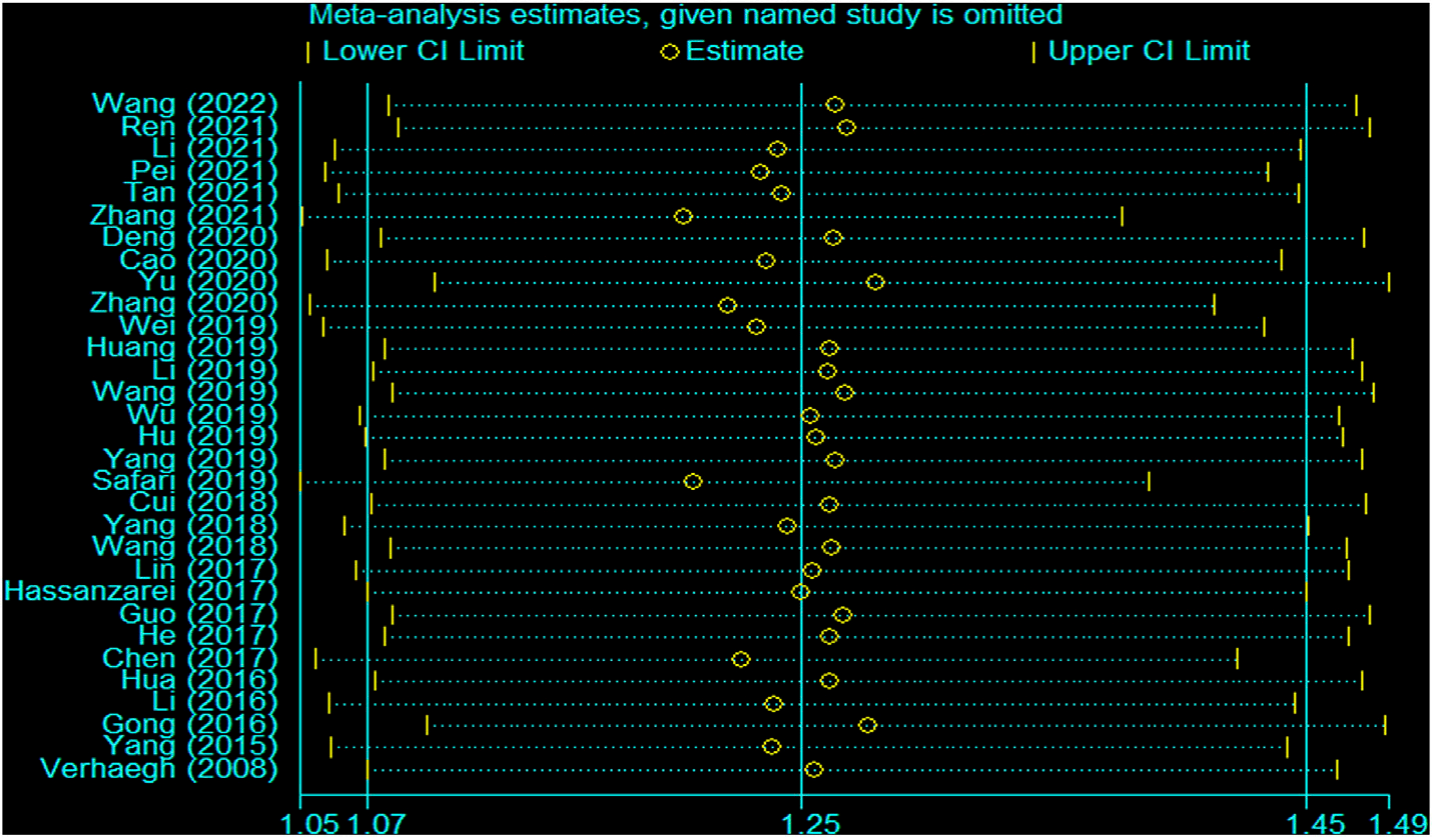
Sensitivity analysis for the H19 rs2839698 polymorphism and cancer susceptibility in the overall population (allele model: A vs G).

**FIGURE 4:**
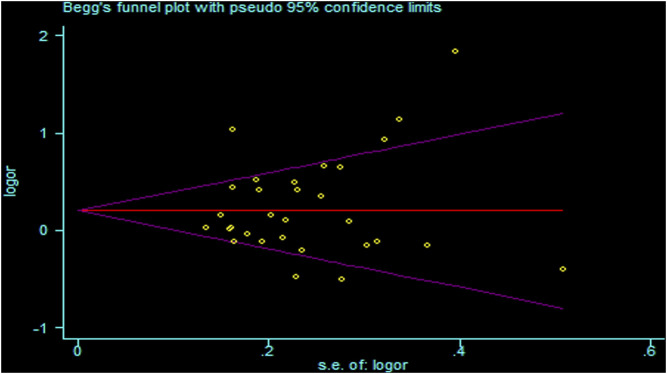
Funnel plots for publication bias of H19 rs2839698 polymorphism in the overall population (allele model: A vs G).

There was heterogeneity in the analysis between these seven SNPs and cancer risk, except for the recessive and homozygous models of rs2107425 and rs3024270, the recessive model of rs3741219, the heterozygous model of rs2735971 as well as the dominant, homozygous and heterozygous models of rs3741216. Therefore, a random-effects model was adopted to analyze the cancer risk. Otherwise, a fixed-effects model was applied for others mentioned above. Sensitivity analyses observed no change of polled OR before and after the removal of each study in all results of all genetic models, suggesting the good stability and reliability of our study (Figure 3). Begg’s funnel plot and Egger’ test were used to evaluate publication bias. The funnel plot was symmetrical, indicating that there was no significant publication bias in the selected literature (Figure 4).

## 4 Discussion

LncRNA plays a crucial role in regulating gene expression and maintaining cell function (Rizk et al., 2022). Accumulating evidence has demonstrated that lncRNA H19 polymorphism was closely related to the initiation, development, and prognosis of cancer (Mitroi et al., 2022; Shaheen et al., 2022). Meanwhile, the study by Allemailem et al. (2021) confirmed the possibility of H19 SNPs in diagnosis and individualized treatment of cancer. A study by Liang et al. (2015) reported that lncRNA H19 can act as the miRNA sponge promoting epithelial to mesenchymal transition (EMT) in CRC. It functions as a primary miRNA precursor or competitive endogenous RNA to regulate specific mRNAs post-transcriptionally (Cai and Cullen, 2007), and the alteration of the SNPs may result in gain and loss of function of miRNA–lncRNA interactions (Correction, 2016). Similarly, alterations in the SNPs may affect the piRNA–lncRNA interactions and thus binding to PIWI proteins that involve in genome integrity, transposon silencing, and epigenetic regulation (Singh et al., 2018; Ozata et al., 2019). Besides, H19 mutation may produce antisense RNA 91H accumulating in breast cancer cells and regulate the expression of insulin growth factor 2 (IGF2) (Berteaux et al., 2008), thereby affecting oncogene and tumor suppressor gene expression. Previously, several meta-analyses have investigated the correlation between H19 polymorphisms and tumorigenesis, and no definitive conclusion has been drawn on this causal relationship (Liu et al., 2020a; Liu et al., 2020b; Wang et al., 2021). Here, we look forward to obtaining more effective evidence to precisely estimate their association and draw a more reliable conclusion. In this study, we collected all the relevant published data up to May 2022 to examine the association between lncRNA H19 rs2839698, rs217727, rs273597, rs2107425, rs3024270, rs3741216, and rs3741219 polymorphisms with cancer risk. We obtained some new conclusions.

Our combined studies demonstrated a clear increase in cancer risk associated with H19 rs2839698 polymorphism in the overall and Asian population group, which contradicts the results of Wang et al. (2021) and Liu et al. (2020b) found no association between the H19 rs2839698 polymorphism and cancer risk neither in the overall group nor in the ethnicity subgroup. The obvious alteration of estimates of effect could be due to increased sample size, given that the 13 newly included studies accounted for 27.18% of individuals. In the subgroup analysis by cancer type, rs2839698 polymorphism was associated with increased cancer risk in hematologic tumors and digestive system tumors. To our knowledge, this is the first meta-analysis to investigate the association between H19 rs2839698 and the risk of hematologic tumors. Moreover, the function of H19 in hematologic tumors is seemingly much more complex than that in other tumor types because of the highly heterogeneous pathogenesis (Pei et al., 2021; Sattarzadeh Bardsiri et al., 2022). We additionally performed a disease-specific meta-analysis on digestive tumors, and the results indicated a significant association between HCC and GC as well as H19 rs2839698 than CRC. The H19 rs2839698 polymorphism effects were tissue-specific, which may give a clue for early screening of the digestive system tumor.

The previous meta-analysis obtained inconsistent findings on the correlation between H19 rs217727 polymorphism and cancer susceptibility. Liu et al. (2020b) found that rs217727 is associated with increased cancer risk in the overall and Asian populations. However, our updated meta-analysis is more supportive of Wang’s study that no effect was detected between rs217727 polymorphism and cancer susceptibility in the general population and ethnicity subgroup (Wang et al., 2021). This difference may result from 18 new articles included in our study. Besides, in the subgroup analysis by cancer type, we found the rs217727 polymorphism may be a risk factor for the occurrence of OSCC and LC, as well as a protective factor for HCC.

In the previous meta-analysis, no association was found between the rs2735971 polymorphism and cancer risk in any genetic models (Li et al., 2020; Wang et al., 2021). However, we added five recently published articles and deleted studies using overlapping data and small sample sizes, finding that the A allele or AA genotype is a potential genetic marker for gastrointestinal tumors. Furthermore, our meta-analysis demonstrated that rs2107425 polymorphism may be a protective factor in the Caucasian population but not the Asian population, which further confirmed the results of the study by Liu et al. (2020b) and Li et al. (2020). The influence of SNP rs2107425 on tumor susceptibility was affected by ethnic factors. We also explored the role of rs3024270, rs3741219, and rs3741216 polymorphisms in cancer risk. No association was found between them.

In addition, the influence of genotypic methods and population information on cancer susceptibility was also explored in our study. When stratified by genotypic methods, rs217727 polymorphism was significantly correlated with PCR-RFLP, rs2839698, and rs3024270 polymorphisms were significantly correlated with TaqMan. Both PCR-RFLP and TaqMan have high confidence in the analysis of gene polymorphism due to their respective merits (Hubáček et al., 2015). In addition, Wang’ meta-analysis pointed out that there was a significant correlation between rs217727 polymorphism and the HB control group when subgroup analysis by a source of controls was performed (Wang et al., 2021). Nevertheless, crucial results were found concentrated in the HB control group in the rs3741219 locus in our study. Insufficient sample size and lack of representativeness may be the reason for this phenomenon.

There are some limitations to this research. Firstly, there is little published research about rs2735971 and rs3741216 polymorphisms, which may limit the statistical power and decrease the reliability of the results. Secondly, in addition to genetic factors, some characteristics of participants, such as age, alcohol intake, and tobacco smoking also influence cancer susceptibility. However, the interaction between these factors and cancer risk could not be assessed due to insufficient data. Thirdly, there exists intensity, moderate or low heterogeneity in this meta-analysis, which may reduce the reliability. Lastly, as mentioned above, because almost all the studies included focused on the Asian population, it is unreasonable to suggest that there is a significant ethnic difference between rs217727 and rs2839698 polymorphisms and cancer risk. This conclusion may not apply to all populations.

In conclusion, our results demonstrated that the H19 rs2839698 polymorphism increased cancer susceptibility, whereas the rs2107425 mutation decreased cancer susceptibility in the Caucasian population. Rs2839698 polymorphism was associated with an increased risk of hematologic tumor and digestive system cancer. Rs217727 polymorphism was associated with OSCC, LC, and HCC risk. Rs2735971 mutation significantly increased the risk of digestive system cancer. Considering the limitations mentioned above, further well-designed case-control studies with larger sample sizes and more different ethnic groups are still warranted to explore the impact of H19 genetic variants on cancer risk.

## Data Availability

The datasets presented in this study can be found in online repositories. The names of the repository/repositories and accession number(s) can be found in the article/Supplementary Material.

## Abbreviations

LncRNA, long non-coding RNA; GWAS, genome-wide association studies; SNP, single-nucleotide polymorphism; CNKI, China National Knowledge Infrastructure; CI, confidence interval; OR, odds ratio; HWE, Hardy–Weinberg equilibrium; PB, population-based; HB, hospital-based; EMT, epithelial to mesenchymal transition; IGF2, insulin growth factor 2.
