# Supplementary material for: Association of lncRNA H19 polymorphisms with cancer susceptibility: An updated meta-analysis based on 53 studies
Source: Front Genet. 2022 Dec 14;13:1051766. doi: 10.3389/fgene.2022.1051766 (PMC9794744; doi:10.3389/fgene.2022.1051766)
Supplement: Supplementary file 1 [file DataSheet2.PDF]

Table 2. Meta-analysis of H19 rs2839698 polymorphism

SNP rs2839698	Number of study	A vs G			AA vs. AG+GG			AA+AG vs. GG			AA vs.GG			AG vs. GG		
		OR(95%CI)	P	I2(%)	OR(95%CI)	P	I2(%)	OR(95%CI)	P	I2(%)	OR(95%CI)	P	I2(%)	OR(95%CI)	P	I2(%)
**Total**	**31**	**1.09 (1.01,1.18)**	**0.034**	**81.2**	1.14 (0.98,1.33)	0.065	75.8	**1.11(1.01,1.21)**	**0.029**	**73.6**	**1.25(1.07,1.46)**	**0.004**	**73.2**	1.06(0.97,1.15)	0.192	66.6
Ethnicity																
**Asian**	**30**	**1.09 (1.01,1.19)**	**0.035**	**81.9**	1.16 (0.99,1.37)	0.088	76.5	**1.12(1.02,1.22)**	**0.020**	**74.0**	**1.26(1.07,1.47)**	**0.004**	**74.1**	1.07(0.98,1.16)	0.128	66.4
Cancer type																
**Hematological tumour**	**2**	**1.64 (1.26,2.13)**	**0.000**	**68.4**	**1.95 (1.49,2.54)**	**0.000**	**6.0**	**1.85(1.41,2.43)**	**0.000**	**43.3**	**2.48(1.74,3.53)**	**0.000**	**30.6**	**1.69(1.37,2.07)**	**0.000**	**0.0**
Nervous system neoplasms	3	1.00 (0.92,1.09)	0.992	0.0	1.03 (0.85,1.26)	0.748	0.0	0.99(0.88,1.11)	0.864	0.0	1.02(0.83,1.26)	0.829	0.0	0.98(0.87,1.11)	0.785	0.0
**Digestive system neoplasm**	7	**1.14 (1.01,1.30)**	**0.04**	**65.8**	**1.31 (1.02,1.69)**	**0.03**	**61.8**	1.13(0.97,1.31)	0.13	58.3	**1.33(1.03,1.74)**	**0.03**	**61.8**	1.06(0.90,1.25)	0.470	58.6
**Hepatocellular cancer**	**3**	**1.15 (1.03,1.29)**	**0.014**	**0.0**	**1.27 (0.94,1.73)**	0.117	34.5	1.17(0.95,1.44)	0.136	45.4	**1.33(1.03,1.72)**	**0.027**	**0.0**	1.12(0.83,1.50)	0.471	70.2
Colorectal cancer	2	0.98 (0.64,1.49)	0.907	91.4	1.04 (0.52,2.09)	0.907	86.2	0.95(0.58,1.55)	0.832	88.2	1.00(0.41,2.45)	0.994	90.6	0.96(0.64,1.42)	0.817	79.6
**Gastric cancer**	**2**	**1.33 (1.13,1.56)**	**0.000**	**0.0**	**1.74 (1.27,2.40)**	**0.001**	**0.0**	**1.27(1.03,1.57)**	**0.024**	**0.0**	**1.76(1.26,2.46)**	**0.001**	**0.0**	1.07(0.75,1.54)	0.699	52.3
**Lung cancer**	**3**	**0.90 (0.81,0.99)**	**0.046**	**0.0**	0.83 (0.62,1.10)	0.186	29.7	0.88(0.77,1.02)	0.082	0.0	0.81(0.64,1.03)	0.083	0.0	0.91(0.75,1.09)	0.310	31.1
Bladder cancer	3	0.98 (0.89,1.09)	0.714	0.0	1.09 (0.88,1.35)	0.429	0.0	0.94(0.82,1.07)	0.335	0.0	1.00(0.80,1.26)	0.988	0.0	0.90(0.75,1.07)	0.228	23.9
Breast cancer	4	0.96 (0.63,1.45)	0.838	94.3	0.91 (0.45,1.83)	0.789	92.7	1.39(0.91,2.15)	0.132	90.8	1.72(0.88,3.34)	0.111	89.3	1.28(0.86,1.90)	0.221	87.4
OSCC	2	0.94 (0.80,1.11)	0.458	0.0	0.66 (0.26,1.70)	0.386	76.5	1.19(0.55,2.58)	0.655	80.9	0.86(0.60,1.22)	0.391	0.0	1.29(0.50,3.33)	0.595	86.6
Cervical cancer	2	1.00 (0.84,1.19)	0.957	0.0	0.85 (0.56,1.29)	0.442	0.0	1.10(0.82,1.47)	0.544	0.0	0.87(0.57,1.34)	0.535	0.0	1.08(0.85,1.36)	0.544	0.0
Source of control																
**PB**	18	**1.14 (1.03,1.26)**	**0.014**	**84.0**	**1.31 (1.12,1.52)**	**0.001**	**68.2**	1.10(0.97,1.25)	0.145	80.5	1.34(1.10,1.64)	0.004	79.1	1.03(0.91,1.15)	0.662	73.3
**HB**	13	1.02 (0.89,1.16)	0.794	77.1	0.91 (0.67,1.23)	0.535	80.0	1.12(0.99,1.26)	0.069	50.5	1.11(0.89,1.39)	0.342	55.4	1.11(0.98,1.26)	0.095	49.6
Methods																
**TaqMan**	19	1.06(1.10,1.13)	0.073	55.4	**1.18(1.02,1.35)**	**0.023**	**57.2**	1.03(0.96,1.11)	0.363	42.6	**1.17(1.02,1.34)**	**0.028**	**52.8**	1.00(0.93,1.08)	1.000	41.1
PCR-RFLP	6	0.93(0.61,1.43)	0.751	93.7	0.83(0.42,1.66)	0.599	92.4	1.27(0.78,2.06)	0.331	89.3	1.25(0.64,2.43)	0.515	88.3	1.22(0.78,1.91)	0.381	85.8
