# Supplementary material for: Association of lncRNA H19 polymorphisms with cancer susceptibility: An updated meta-analysis based on 53 studies
Source: Front Genet. 2022 Dec 14;13:1051766. doi: 10.3389/fgene.2022.1051766 (PMC9794744; doi:10.3389/fgene.2022.1051766)
Supplement: Supplementary file 2 [file DataSheet4.PDF]

Table 4 .Meta-analysis of H19 rs2107425 and rs2735971 polymorphisms

**SNP rs2107425**	**Number**	**T vs. C**			**TT vs. TC+CC**			**TT+TC vs. CC**			**TT vs.CC**			**TC vs. CC**		
	**of study**	**OR(95%CI)**	**P**	**I^2(%)**	**OR(95%CI)**	**P**	**I^2(%)**	**OR(95%CI)**	**P**	**I^2(%)**	**OR(95%CI)**	**P**	**I^2(%)**	**OR(95%CI)**	**P**	**I^2(%)**
Total	13	0.96 (0.90, 1.03)	0.236	58.0	1.01 (0.94,1.09)	0.938	38.4	0.95 (0.86,1.04)	0.259	62.5	0.97 (0.90,1.05)	0.494	45.9	0.95 (0.85,1.05)	0.286	63.5
Ethnicity																
Asian	7	1.01 (0.90, 1.13)	0.915	47.9	0.97 (0.74,1.40)	0.619	54.9	1.05 (0.93,1.19)	0.413	10.5	0.98 (0.76,1.26)	0.873	54.8	1.08 (0.96,1.22)	0.249	0.0
**Caucasian**	**5**	**0.90 (0.84, 0.97)**	**0.006**	**51.8**	1.01 (0.92,1.10)	0.848	8.1	**0.84 (0.75,0.94)**	**0.003**	**65.4**	0.93 (0.83,1.04)	0.177	13.9	**0.82 (0.72,0.94)**	**0.003**	**69.1**
Cancer type																
Bladder cancer	2	1.00 (0.81, 1.23)	0.971	29.7	1.00 (0.72,1.37)	0.985	0.0	0.95 (0.57,1.58)	0.842	77.2	1.05 (0.74,1.50)	0.783	0.0	0.93 (0.49,1.76)	0.815	83.6
Lung cancer	2	1.15 (0.92, .44)	0.226	56.0	1.24 (0.95,1.63)	0.114	43.7	1.21 (0.95,1.54)	0.119	21.0	1.30(0.81,2.09)	0.280	56.1	1.17 (0.94,1.47)	0.168	0.0
Breast cancer	3	0.95 (0.80, 1.13)	0.567	77.7	0.95 (0.76,1.20)	0.675	51.8	0.94 (0.75,1.20)	0.630	77.2	0.94 (0.67,1.31)	0.715	73.0	0.94 (0.75,1.19)	0.617	73.2
Ovarian cancer	2	0.92 (0.80, 1.05)	0.196	82.5	1.05 (0.95,1.16)	0.370	0.0	0.85 (0.68,1.05)	0.127	88.6	0.98 (0.86,1.11)	0.698	18.4	0.82 (0.65,1.04)	0.102	89.2
Cervical cancer	2	0.95 (0.81, 1.12)	0.532	0.0	0.91 (0.67,1.22)	0.511	0.0	0.95 (0.76,1.21)	0.696	0.0	0.90 (0.65, 1.25)	0.534	0.0	0.98 (0.76,1.25)	0.848	0.0
Source of control																
HB	5	0.93 (0.84 1.02)	0.120	0.0	0.81(0.67,0.99)	0.036	29.8	0.95 (0.83,1.10)	0.500	0.0	0.82 (0.67,1.04)	0.073	0.0	0.98 (0.83,1.16)	0.805	0.0
PB	8	0.97 (0.89, 1.07)	0.568	73.3	1.04 (0.96,1.13)	0.335	22.8	0.95 (0.83,1.09)	0.427	77.4	1.01 (0.87,1.18)	0.880	55.3	0.93 (0.81,1.07)	0.302	76.4
Methods																
TaqMan	6	0.93 (0.85, 1.01)	0.062	45.7	0.93(0.79,1.09)	0.362	51.7	0.92 (0.81,1.04)	0.196	63.3	0.95 (0.87,1.04)	0.292	31.7	0.94 (0.81,1.09)	0.417	71.8

**SNP rs2735971**	**Number**	**T vs. C**			**TT vs. TC+CC**			**TT+TC vs.CC**			**TT vs.CC**			**TC vs. CC**		
	**of study**	**OR(95%CI)**	**P**	**I^2(%)**	**OR(95%CI)**	**P**	**I^2(%)**	**OR(95%CI)**	**P**	**I^2(%)**	**OR(95%CI)**	**P**	**I^2(%)**	**OR(95%CI)**	**P**	**I^2(%)**
Total	8	1.00(0.86,1.15)	0.948	70.7	1.09(0.83,1.44)	0.527	59.2	0.96(0.82,1.12)	0.591	52.6	1.16(0.82,1.64)	0.406	65.1	0.91(0.82,1.00)	0.053	12.4
Cancer type																
**b Digestive system neoplasm**	3	**0.88(0.78,0.98)**	**0.021**	**0.0**	0.86(0.65,1.13)	0.284	0.0	**0.85(0.74,0.98)**	**0.024**	**0.0**	0.87(0.64,1.18)	0.371	0.0	**0.86(0.74,0.99)**	**0.034**	**0.0**
Bladder cancer	2	1.01(0.89,1.16)	0.837	0.0	1.45(0.82,2.54)	0.199	21.7	0.97(0.83,1.13)	0.700	0.0	1.38(0.83,2.30)	0.209	13.8	0.93(0.79,1.10)	0.400	0.0
Source of control																
PB	4	1.00(0.75,1.35)	0.981	85.9	1.03(0.64,1.66)	0.901	78.1	0.99(0.73,1.34)	0.934	75.1	1.06(0.60,1.88)	0.839	81.5	0.89(0.77,1.01)	0.078	47.4
HB	4	1.00(0.90,1.12)	0.966	0.0	1.15(0.90,1.48)	0.260	0.0	0.96(0.84,1.10)	0.554	0.0	1.28(0.93,1.77)	0.135	0.0	0.93(0.81,1.07)	0.335	0.0
Methods																
TaqMan	6	1.06(0.88,1.27)	0.566	75.3	1.20(0.87,1.66)	0.258	63.6	1.04(0.84,1.28)	0.743	67.1	1.34(0.90,1.99)	0.150	64.8	0.93(0.83,1.04)	0.189	33.0
