# Supplementary material for: Association of lncRNA H19 polymorphisms with cancer susceptibility: An updated meta-analysis based on 53 studies
Source: Front Genet. 2022 Dec 14;13:1051766. doi: 10.3389/fgene.2022.1051766 (PMC9794744; doi:10.3389/fgene.2022.1051766)
Supplement: Supplementary file 3 [file DataSheet3.PDF]

Table 3. Meta-analysis of H19 rs217727 polymorphism

SNP rs217727	Number of study	A vs. G			AA vs. AG+GG			AA+AG vs. GG			AA vs. GG			AG vs. GG		
		OR(95%CI)	P	I2(%)	OR(95%CI)	P	I2(%)	OR(95%CI)	P	I2(%)	OR(95%CI)	P	I2(%)	OR(95%CI)	P	I2(%)
Total	36	1.07(0.99,1.14)	0.074	77.6	1.09(0.97,1.24)	0.144	67.6	1.07(0.98,1.17)	0.114	74.1	1.11(0.97,1.27)	0.093	70.0	1.05(0.96,1.14)	0.235	68.6
Ethnicity																
Asian	33	1.07(1.00,1.15)	0.056	77.8	1.11(0.98,1.25)	0.104	68.9	1.08(0.99,1.19)	0.092	74.6	1.14(0.99,1.30)	0.068	70.6	1.06(0.97,1.16)	0.198	69.9
Caucasian	2	0.85(0.71,1.01)	0.058	0.0	0.69(0.43,1.12)	0.134	0.0	0.84(0.68,1.03)	0.100	0.0	0.66(0.40,1.08)	0.095	0.0	0.87(0.70,1.08)	0.199	0.0
Cancer type																
Bladder cancer	4	1.02(0.89,1.16)	0.819	38.2	0.73(0.40,1.32)	0.292	79.9	1.11(0.80,1.52)	0.543	78.9	0.89(0.59,1.35)	0.592	54.1	1.16(0.78,1.73)	0.461	85.3
Breast cancer	7	1.18(0.92,1.50)	0.198	88.1	1.33(0.87,2.03)	0.189	77.2	1.14(0.84,1.55)	0.394	86.1	1.37(0.84,2.23)	0.209	80.4	1.05(0.79,1.41)	0.724	82.5
**OSCC**	**3**	**1.31(1.14,1.50)**	**0.000**	**21.8**	**1.67(1.04,2.67)**	**0.034**	**64.2**	**1.37(1.16,1.60)**	**0.000**	**0.0**	**1.89(1.19,2.99)**	**0.007**	**56.7**	**1.27(1.07,1.51)**	**0.005**	**0.0**
**Lung cancer**	**3**	**1.16(1.06,1.27)**	**0.002**	**0.0**	**1.31(1.03,1.66)**	**0.028**	**44.7**	**1.16(1.01,1.33)**	**0.031**	**0.0**	**1.38(1.14,1.67)**	**0.001**	**0.0**	1.09(0.95,1.26)	0.219	0.0
Cervical cancer	3	1.33(0.97,1.81)	0.078	75.5	1.54(0.90,2.62)	0.113	56.4	1.37(0.98,1.93)	0.068	65.5	1.73(0.90,3.33)	0.099	68.9	1.30(0.98,1.73)	0.066	43.8
Gastric cancer	4	1.02(0.70,1.48)	0.916	89.8	1.00(0.51,1.93)	0.988	87.6	1.09(0.77,1.55)	0.619	77.4	1.10(0.53,2.27)	0.793	87.6	1.14(0.91,1.43)	0.246	37.8
**Hepatocellular cancer**	2	0.79(0.60,1.05)	0.100	71.7	**0.73(0.54,1.00)**	**0.048**	**0.0**	0.75(0.47,1.21)	0.237	83.8	**0.68(0.49,0.93)**	**0.017**	**0.0**	0.77(0.44,1.34)	0.359	86.3
Nervous system neoplasms	3	1.00(0.91,1.09)	0.918	0.0	1.06(0.88,1.27)	0.562	0.0	0.97(0.86,1.09)	0.612	0.0	1.03(0.85,1.25)	0.737	0.0	0.96(0.85,1.08)	0.465	0.0
Source of control																
PB	25	1.05(0.97,1.13)	0.252	74.1	1.09(0.95,1.24)	0.222	66.6	1.05(0.95,1.16)	0.336	70.6	1.11(0.96,1.28)	0.166	65.8	1.03(0.94,1.14)	0.500	67.2
HB	11	1.14(0.96,1.34)	0.135	84.2	1.17(0.87,1.57)	0.299	72.5	1.15(0.94,1.41)	0.176	81.2	1.27(0.89,1.79)	0.186	78.4	1.11(0.92,1.37)	0.275	73.7
Methods																
TaqMan	19	0.98(0.91,1.04)	0.474	65.0	0.97(0.85,1.11)	0.623	65.4	0.98(0.89,1.07)	0.594	62.5	0.97(0.85,1.10)	0.713	59.5	0.99(0.90,1.09)	0.754	62.6
**PCR-RFLP**	**7**	**1.46(1.09,1.97)**	**0.012**	**80.3**	**2.02(1.07,3.85)**	**0.031**	**67.5**	**1.51(1.07,2.14)**	**0.019**	**76.0**	**2.47(1.16,5.27)**	**0.045**	**78.9**	**1.37(1.03,1.83)**	**0.033**	**62.3**
MassARRAY	3	1.24(0.99,1.54)	0.056	75.3	1.43(0.96,2.13)	0.083	64.1	1.26(0.99,1.59)	0.061	63.8	1.57(0.97,2.53)	0.066	72.3	1.18(0.98,1.43)	0.090	39.3
