# Supplementary material for: Association of lncRNA H19 polymorphisms with cancer susceptibility: An updated meta-analysis based on 53 studies
Source: Front Genet. 2022 Dec 14;13:1051766. doi: 10.3389/fgene.2022.1051766 (PMC9794744; doi:10.3389/fgene.2022.1051766)
Supplement: Supplementary file 4 [file DataSheet1.PDF]

Table 1. Characteristics of the studies included in the meta-analysis

First author	Year	Country	Ethnicity	Cancer type	Case/control	Case			Control			Source of control	Genotyping methods	HWE
					rs217727 G>A	GG	GA	AA	GG	GA	AA			
Azab	2022	Egypt	African	Breast cancer	100/50	37	46	17	28	19	3	HB	PCR-RFLP	0.93
Li	2021	Mixed	Asian	Wilms tumor	355/1068	177	130	48	486	469	113	PB	TaqMan	0.99
Pei	2021	Mixed	Asian	Leukemia	266/266	111	120	35	114	120	32	PB	PCR-RFLP	0.96
Tan	2021	Mixed	Asian	Hepatocellular cancer	213/957	126	68	19	438	410	109	HB	TaqMan	0.38
Ying	2020	China	Asian	Gastric cancer	128/130	39	63	26	58	58	14	PB	PCR-RFLP	0.93
Deng	2020	China	Asian	Glioma	605/1300	254	278	73	557	591	152	PB	MassARRAY	0.80
Ghapanchi	2020	Iran	Asian	Oral squamous cell carcinoma	200/200	110	75	15	133	64	3	HB	ARMS-PCR	0.65
Petkevicius	2020	Lithuania	Caucasian	Gastric cancer	610/473	352	229	29	259	184	30	HB	TaqMan	0.72
Cao	2020	China	Asian	Renal cell carcinoma	1027/1094	350	494	183	343	550	201	PB	TaqMan	0.45
Wei	2019	China	Asian	Gastric cancer	225/200	88	72	65	63	44	93	PB	TaqMan	<0.01
Huang	2019	China	Asian	Cervical cancer	235/325	102	103	28	135	139	39	HB	TaqMan	0.73
Li	2019	Mixed	Asian	Neuroblastoma	700/1516	331	289	80	679	674	161	PB	TaqMan	0.74
Wang	2019	China	Asian	Lung cancer	564/1536	162	277	125	493	751	291	PB	TaqMan	0.33
Wu	2019	China	Asian	Hepatocellular cancer	359/1190	154	170	35	495	539	156	HB	TaqMan	0.63
Hu	2019	Mixed	Asian	Neuroblastoma	393/810	186	164	43	382	342	86	PB	TaqMan	0.47
Yang	2019	China	Asian	Bladder cancer	431/431	185	202	44	191	188	52	PB	TaqMan	0.58
Abdollahzadeh	2019	Iran	Asian	Breast cancer	150/100	116	29	5	86	14	0	PB	PCR-RFLP	0.45
Safari	2019	Iran	Asian	Breast cancer	111/130	79	30	2	64	54	12	PB	4P-ARMSPCR	0.90
Yuan	2018	China	Asian	Oral squamous cell carcinoma	431/984	186	194	51	488	423	73	PB	MassARRAY	0.00
Cui	2018	China	Asian	Breast cancer	1492/1677	613	694	185	686	774	217	PB	TaqMan	0.96
Li	2018	China	Asian	Lung cancer	555/618	210	250	95	246	305	67	PB	TaqMan	0.05
Yin	2018	China	Asian	Lung cancer	556/395	204	264	88	165	172	58	PB	Illumina	0.23
Li	2018	China	Asian	Bladder cancer	200/200	51	140	9	84	90	26	PB	TaqMan-MGB	0.71
Guo	2017	China	Asian	Oral squamous cell carcinoma	362/740	101	181	80	255	348	137	PB	Illumina	0.34
He	2017	China	Asian	Osteosarcoma	193/383	79	102	12	195	165	23	HB	TaqMan	0.12
He	2017	China	Asian	Cervical cancer	250/250	118	104	28	149	87	14	PB	PCR-RFLP	0.78
Chen	2017	China	Asian	Prostate cancer	145/162	71	48	26	65	63	34	PB	TaqMan	0.01
Hu	2017	China	Asian	Pancreatic cancer	416/416	133	200	83	128	196	92	HB	TaqMan	0.30
Lin	2017	China	Asian	Breast cancer	1005/1020	452	440	113	484	432	104	PB	Plex SNP scan	0.60
Hassanzarei	2017	Iran	Asian	Breast cancer	230/240	71	132	27	125	113	2	HB	PCR-RFLP	<0.01
Hua	2016	China	Asian	Bladder cancer	1046/1394	431	467	148	573	665	156	HB	TaqMan	0.07
Li	2016	China	Asian	Colorectal cancer	1147/1203	480	514	153	456	570	177	PB	TaqMan	0.91
Jin	2016	China	Asian	Cervical cancer	246/284	117	103	26	169	99	16	PB	MassARRAY	0.77
Xia	2016	China	Asian	Breast cancer	464/467	160	156	148	139	212	116	PB	CRS-RFLP	0.05
Yang	2015	China	Asian	Gastric cancer	500/500	160	252	88	193	244	63	HB	TaqMan	0.30
Verhaegh	2008	Netherlands	Caucasian	Bladder cancer	177/204	114	59	4	115	80	9	PB	PCR-RFLP	0.29
					rs2839698 G>A	GG	GA	AA	GG	GA	AA			
Wang	2022	China	Asian	Cervical cancer	345/360	179	144	22	195	137	28	HB	TaqMan	0.567
Ren	2021	China	Asian	Lung cancer	452/463	260	152	40	239	179	45	HB	TaqMan	0.266
Li	2021	Mixed	Asian	Wilms tumor	355/1068	174	127	54	488	480	100	PB	TaqMan	0.245
Pei	2021	Mixed	Asian	Leukemia	266/266	91	131	44	119	117	30	PB	PCR-RFLP	0.88

Tan	2021	Mixed	Asian	**Hepatocellular cancer**	213/957	102	78	33	439	424	94	HB	TaqMan	0.57
Zhang	2021	China	Asian	Lymphoma	573/688	192	244	137	351	248	89	PB	PCR-RFLP	<0.01
Deng	2020	China	Asian	Glioma	605/1300	311	240	54	675	504	121	PB	MassARRAY	0.06
Cao	2020	China	Asian	Renal cell carcinoma	1027/1094	516	435	76	615	425	54	PB	TaqMan	0.07
Yu	2020	China	Asian	**Colorectal cancer**	315/441	134	140	40	154	211	74	PB	PCR-RFLP	0.91
Zhang	2020	China	Asian	Ovarian cancer	203/196	70	93	38	92	88	16	HB	MassARRAY	0.43
Wei	2019	China	Asian	**Gastric cancer**	225/200	90	68	67	88	78	34	PB	TaqMan	0.15
Huang	2019	China	Asian	Cervical cancer	235/325	115	99	20	154	134	30	HB	qPCR	0.91
Li	2019	Mixed	Asian	Neuroblastoma	700/1516	331	300	69	704	667	143	PB	TaqMan	0.41
Wang	2019	China	Asian	Lung cancer	564/1536	277	225	61	712	645	175	PB	TaqMan	0.60
Wu	2019	China	Asian	**Hepatocellular cancer**	359/1190	140	178	41	532	524	134	HB	TaqMan	0.77
Hu	2019	Mixed	Asian	Neuroblastoma	393/810	179	175	39	365	373	72	PB	TaqMan	0.09
Yang	2019	China	Asian	Bladder cancer	431/431	206	170	55	192	184	55	PB	TaqMan	0.30
Safari	2019	Iran	Asian	Breast cancer	111/130	15	57	39	53	55	22	PB	4P-ARMSPCR	0.24
Cui	2018	China	Asian	Breast cancer	1492/1677	802	568	122	875	673	129	PB	TaqMan	0.98
Yang	2018	China	Asian	**Hepatocellular cancer**	466/462	215	211	40	245	185	32	HB	KASP	0.71
Wang	2018	China	Asian	Oral squamous cell carcinoma	124/121	20	96	8	32	70	19	HB	PCR-RFLP	0.06
Lin	2017	China	Asian	Breast cancer	1005/1020	452	440	113	484	432	104	PB	Plex SNP scan	0.60
Hassanzarei	2017	Iran	Asian	Breast cancer	230/240	0	64	166	0	18	222	HB	PCR-RFLP	0.55
Guo	2017	China	Asian	Oral squamous cell carcinoma	362/741	133	171	58	244	377	120	PB	Illumina	0.20
He	2017	China	Asian	Osteosarcoma	193/383	83	98	12	178	175	30	HB	TaqMan	0.15
Chen	2017	China	Asian	Prostate cancer	145/162	48	56	41	68	71	23	PB	TaqMan	0.52
Hua	2016	China	Asian	Bladder cancer	1049/1397	552	418	79	729	565	103	HB	TaqMan	0.65
Li	2016	China	Asian	**Colorectal cancer**	1147/1203	583	462	102	666	462	75	PB	TaqMan	0.67
Gong	2016	China	Asian	Lung cancer	496/206	237	220	39	99	80	27	HB	MassARRAY	0.10
Yang	2015	China	Asian	**Gastric cancer**	500/500	250	195	55	284	178	38	HB	TaqMan	0.18
Verhaegh	2008	Netherlands	Caucasian	Bladder cancer	177/204	54	74	49	52	109	43	PB	PCR-RFLP	0.31
					rs2107425 C>T	CC	CT	TT	CC	CT	TT			
Wang	2022	China	Asian	Cervical cancer	345/360	114	173	58	120	168	72	HB	TaqMan	0.35
Ghapanchi	2020	Iran	Asian	Oral squamous cell carcinoma	200/200	79	94	27	74	101	25	HB	ARMS-PCR	0.29
Yang	2019	China	Asian	Bladder cancer	431/431	152	213	66	171	190	70	PB	TaqMan	0.16
Huang	2019	China	Asian	Cervical cancer	235/325	88	107	38	109	155	48	HB	TaqMan	0.56
Wu	2019	China	Asian	Hepatocellular cancer	359/1190	134	185	40	422	560	208	HB	TaqMan	0.35
Yin	2018	China	Asian	Lung cancer	556/395	161	266	129	140	185	70	PB	Illumina	0.51
Gong	2016	China	Asian	Lung cancer	479/203	181	235	63	79	96	28	HB	MassARRAY	0.89
Butt	2012	Sweden	Caucasian	Breast cancer	678/1355	360	250	68	637	573	145	PB	MassARRAY	0.34
Barnholtz	2010	USA	Caucasian	Breast cancer	1225/1118	604	516	105	521	478	119	PB	GoldenGateassay	0.55
Barnholtz	2010	USA	African	Breast cancer	737/658	161	390	186	170	339	149	PB	GoldenGate assay	0.42
Quaye	2009	Mixed	Caucasian	Ovarian cancer	1460/2463	767	544	149	1118	1098	247	PB	TaqMan	0.35
Song	2009	Mixed	Caucasian	Ovarian cancer	5366/8538	2619	2192	555	4029	3667	842	PB	TaqMan	0.86
Verhaegh	2008	Netherlands	Caucasian	Bladder cancer	177/204	92	65	20	89	96	19	PB	PCR-RFLP	0.34
					rs3024270 G>C	GG	CG	CC	GG	CG	CC			
Li	2021	Mixed	Asian	Wilms tumor	355/1068	94	141	120	222	556	290	PB	TaqMan	0.14
Tan	2021	Mixed	Asian	Hepatocellular cancer	213/957	76	87	50	204	489	264	HB	TaqMan	0.42
Wei	2020	China	Asian	Gastric cancer	115/115	13	40	62	19	59	37	PB	TaqMan	0.58
Huang	2019	China	Asian	Cervical cancer	235/325	60	120	51	95	150	71	HB	qPCR	0.42

Li	2019	Mixed	Asian	Neuroblastoma	700/1514	154	362	184	318	781	415	PB	TaqMan	0.16
Wu	2019	China	Asian	Hepatocellular cancer	359/1190	85	187	87	263	593	334	HB	TaqMan	1.00
Hu	2019	Mixed	Asian	Neuroblastoma	393/810	91	203	99	173	424	213	PB	TaqMan	0.16
Yang	2019	China	Asian	Bladder cancer	431/431	107	210	114	103	208	120	PB	TaqMan	0.49
Yang	2018	China	Asian	Hepatocellular cancer	471/466	151	225	95	170	215	81	HB	KASP	0.36
Li	2018	China	Asian	Bladder cancer	200/200	83	101	16	81	97	22	PB	TaqMan-MGB	0.38
Guo	2017	China	Asian	Oral squamous cell carcinoma	362/740	104	183	75	245	350	145	PB	Illumina	0.32
He	2017	China	Asian	Osteosarcoma	193/383	85	91	17	173	179	31	HB	TaqMan	0.10
Chen	2017	China	Asian	Prostate cancer	145/162	20	46	79	27	59	76	PB	TaqMan	0.01
Hua	2016	China	Asian	Bladder cancer	1047/1395	346	527	174	447	688	260	HB	TaqMan	0.87
Li	2016	China	Asian	Colorectal cancer	1147/1203	235	527	385	201	582	420	PB	TaqMan	0.98
					rs3741219 T>C	TT	TC	CC	TT	TC	CC			
Wei	2020	China	Asian	Gastric cancer	115/115	41	53	21	37	54	24	PB	TaqMan	0.60
Deng	2020	China	Asian	Glioma	605/1300	439	107	59	651	520	129	PB	MassARRAY	0.10
Farzaneh	2020	Iran	Asian	Uterine leiomyomas	155/204	57	73	25	74	95	35	HB	PCR-RFLP	0.63
Cao	2020	China	Asian	Renal cell carcinoma	1027/1094	552	389	86	567	416	111	PB	TaqMan	0.01
Zhang	2020	China	Asian	Ovarian cancer	211/195	190	1	20	186	0	9	HB	MassARRAY	<0.01
Abdollahzadeh	2019	Iran	Asian	Breast cancer	150/100	119	24	7	80	17	3	PB	PCR-RFLP	0.10
Yang	2019	China	Asian	Bladder cancer	431/431	192	181	58	185	190	56	PB	TaqMan	0.51
Wu	2019	China	Asian	Hepatocellular cancer	359/1190	129	182	48	517	536	137	HB	TaqMan	0.91
Cui	2018	China	Asian	Breast cancer	2881/3220	782	582	127	832	706	139	PB	TaqMan	0.53
Wang	2018	China	Asian	Oral squamous cell carcinoma	119/114	56	54	9	47	59	8	HB	PCR-RFLP	0.07
Hassanzarei	2017	Iran	Asian	Breast cancer	230/240	63	126	41	109	102	29	HB	PCR-RFLP	0.50
Xia	2016	China	Asian	Breast cancer	464/467	238	186	40	245	182	40	PB	CRS-RFLP	0.46
Yang	2015	China	Asian	Gastric cancer	500/500	260	187	53	268	189	43	HB	TaqMan	0.25
					rs3741216 A>T	AA	AT	TT	AA	AT	TT			
Cao	2020	China	Asian	Renal cell carcinoma	1027/1094	728	264	35	791	255	48	PB	TaqMan	<0.01
Wei	2019	China	Asian	Gastric cancer	225/200	79	91	55	70	78	52	PB	TaqMan	0.003
Hassanzarei	2017	Iran	Asian	Breast cancer	230/240	0	26	204	0	65	175	HB	PCR-RFLP	0.02
Yang	2015	China	Asian	Gastric cancer	500/500	380	102	18	379	109	12	HB	TaqMan	0.22
					rs2735971 C>T	CC	CT	TT	CC	CT	TT			
Wei	2019	China	Asian	Gastric cancer	225/200	60	81	84	48	62	90	PB	TaqMan	<0.01
Li	2018	China	Asian	Bladder cancer	200/200	128	62	10	126	70	4	HB	TaqMan	0.10
Yang	2018	China	Asian	Hepatocellular cancer	465/465	327	126	12	313	139	13	HB	KASP	0.70
Guo	2017	China	Asian	Oral squamous cell carcinoma	361/739	191	141	29	351	308	80	PB	Illumina	0.32
He	2017	China	Asian	Osteosarcoma	193/383	11	94	88	32	182	169	HB	TaqMan	0.08
Chen	2017	China	Asian	Prostate cancer	145/162	39	55	51	69	61	32	PB	TaqMan	0.01
Li	2016	China	Asian	Colorectal cancer	1147/1203	773	334	40	765	398	40	PB	TaqMan	0.11
Hua	2016	China	Asian	Bladder cancer	1049/1396	704	302	43	928	422	46	HB	TaqMan	0.82
