# Supplementary material for: Association of lncRNA H19 polymorphisms with cancer susceptibility: An updated meta-analysis based on 53 studies
Source: Front Genet. 2022 Dec 14;13:1051766. doi: 10.3389/fgene.2022.1051766 (PMC9794744; doi:10.3389/fgene.2022.1051766)
Supplement: Supplementary file 5 [file DataSheet5.PDF]

Table5. Meta-analysis of H19 rs3024270, rs3741219 and rs3741216 polymorphisms

**SNP rs3024270**	**Number of study**	**C vs. G**			**CC vs. CG+GG**			**CC+GC vs. GG**			**CC vs.GG**			**CGvs.GG**		
		**OR(95%CI)**	**P**	**I^2(%)**	**OR(95%CI)**	**P**	**I^2(%)**	**OR(95%CI)**	**P**	**I^2(%)**	**OR(95%CI)**	**P**	**I^2(%)**	**OR(95%CI)**	**P**	**I^2(%)**
Total	15	0.99(0.92,1.06)	0.753	57.3	0.99(0.92,1.07)	0.810	48.5	0.94(0.83,1.06)	0.309	59.2	0.92(0.84,1.00)	0.058	46.1	0.93(0.81,1.05)	0.24	59.4
Cancer type																
Hepatocellular cancer	3	0.90(0.68,1.19)	0.452	84.9	0.91(0.76,1.09)	0.313	47.0	0.82(0.49,1.37)	0.448	89.2	0.82(0.49,1.38)	0.450	83.1	0.83(0.50,1.37)	0.46	87.7
Neuroblastoma	2	0.95(0.86,1.06)	0.354	0.0	0.94(0.80,1.11)	0.491	0.0	0.93(0.78,1.10)	0.395	0.0	0.90(0.73,1.11)	0.341	0	0.94(0.78,1.13)	0.51	0
Bladder cancer	3	0.94(0.86,1.03)	0.199	0.0	0.88(0.74,1.04)	0.125	0.0	0.96(0.83,1.10)	0.519	0.0	0.87(0.71,1.05)	0.141	0	0.99(0.85,1.15)	0.89	0
Source of control																
PB	9	1.01(0.92,1.11)	0.788	52.5	1.09(0.92,1.27)	0.327	60.3	0.93(0.82,1.00)	0.279	35.0	0.96(0.83,1.12)	0.624	32.6	0.90(0.78,1.05)	0.18	43.6
HB	6	0.95(0.83,1.09)	0.463	67.4	0.91(0.80,1.03)	0.150	0.0	0.93(0.73,1.19)	0.575	77.2	0.90(0.69,1.17)	0.419	63.6	0.95(0.74,1.20)	0.66	74.3
Methods																
TaqMan	12	0.97(0.89,1.05)	0.415	57.4	1.01(0.89,1.15)	0.869	55.3	0.89(0.78,1.01)	0.079	55.9	**0.88(0.79,0.97)**	**0.009**	**40.7**	0.87(0.76,1.01)	0.06	58.5

**SNP rs3741219**	**Number of study**	**C vs. T**			**CC vs. TC+TT**			**CC+TC vs.TT**			**CC vs.TT**			**TC vs. TT**		
		**OR(95%CI)**	**P**	**I^2(%)**	**OR(95%CI)**	**P**	**I^2(%)**	**OR(95%CI)**	**P**	**I^2(%)**	**OR(95%CI)**	**P**	**I^2(%)**	**OR(95%CI)**	**P**	**I^2(%)**
Total	13	1.02(0.87,1.19)	0.843	85.5	1.05(0.93,1.18)	0.422	0.0	1.00(0.79,1.27)	0.999	88.8	1.07(0.88,1.30)	0.487	53.1	0.94(0.73,1.22)	0.645	89.0
Cancer type																
b Digestive system neoplasm	3	1.12(0.97,1.30)	0.134	24.8	1.15(0.90,1.48)	0.264	0.0	1.15(0.91,1.45)	0.240	41.0	1.25 (0.10,1.64)	0.098	0.0	1.13(0.89,1.44)	0.302	37.9
Reproductive	2	1.41(0.63,3.19)	0.403	84.3	1.35(0.59,3.06)	0.481	64.6	1.39(0.61,3.16)	0.428	69.7	1.36(0.60,3.13)	0.467	62.7	1.02(0.65,1.61)	0.933	0.0
Breast cancer	4	1.14(0.88,1.47)	0.322	80.2	1.10(0.90,1.35)	0.332	0.0	1.19(0.82,1.73)	0.356	84.1	1.30(0.83,2.04)	0.253	65.8	1.16(0.80,1.68)	0.439	82.0
Source of control																
PB	7	0.88(0.73,1.05)	0.158	84.8	0.96(0.84,1.11)	0.585	0.0	0.82(0.61,1.10)	0.177	90.1	0.87(0.75,1.01)	0.064	0.0	0.79(0.56,1.11)	0.175	91.7
HB	6	**1.22(1.00,1.49)**	**0.046**	**67.3**	**1.27(1.03,1.56)**	**0.027**	**0.0**	1.29(0.97,1.72)	0.079	70.3	**1.44(1.15,1.79)**	**0.011**	**31.1**	1.21(0.91,1.61)	0.187	63.5
Methods																
TaqMan	6	1.00(0.91,1.10)	0.984	47.7	1.01(0.88,1.17)	0.870	0.0	1.00(0.87,1.14)	0.970	50.7	1.02(0.85,1.22)	0.864	26.4	0.99(0.87,1.13)	0.887	43.9
PCR-RFLP	4	1.14(0.82,1.58)	0.431	69.8	1.24(0.88,1.75)	0.222	0.0	1.18(0.72,1.94)	0.519	76.7	1.40(0.79,2.48)	0.254	85.3	1.15(0.70,1.88)	0.590	73.8

**SNP rs3741216**	**Number of study**	**T vs. A**			**TT vs. TA+AA**			**TT+TA vs.AA**			**TT vs.AA**			**TA vs. AA**		
		**OR(95%CI)**	**P**	**I^2(%)**	**OR(95%CI)**	**P**	**I^2(%)**	**OR(95%CI)**	**P**	**I^2(%)**	**OR(95%CI)**	**P**	**I^2(%)**	**OR(95%CI)**	**P**	**I^2(%)**
Total	4	1.20(0.89,1.60)	0.234	79.4	1.31(0.70,2.45)	0.394	83.2	1.04(0.90,1.20)	0.605	0.0	0.94(0.69,1.27)	0.681	2.9	1.06(0.91,1.24)	0.465	0.0
Cancer type																
Gastric cancer	2	1.01(0.84,1.21)	0.944	0.0	1.05(0.72,1.53)	0.748	22.9	0.99(0.78,1.25)	0.941	0.0	1.09(0.72,1.64)	0.697	4.7	0.97(0.75,1.24)	0.779	0.0
Source of control																
PB	2	1.00(0.87,1.15)	0.968	0.0	0.84(0.62,1.15)	0.281	0.0	1.06(0.89,1.26)	0.520	0.0	0.85(0.61,1.19)	0.350	0.0	1.11(0.93,1.33)	0.265	0.0
HB	2	1.62(0.66,3.97)	0.296	91.0	2.23(1.19,4.19)	0.012	51.2	0.99(0.74,1.32)	0.941	0.0	1.50(0.71,3.15)	0.289	0.0	0.93(0.69,1.27)	0.658	0.0
